# Preparation, Characterization, Solubility, and Antioxidant Capacity of Ellagic Acid-Urea Complex

**DOI:** 10.3390/ma15082836

**Published:** 2022-04-12

**Authors:** Hitomi Sakurai, Mitsuaki Suzuki, Shoko Itakura, Hiroaki Todo, Florencio Arce, Gerard Lee See, Takashi Tanikawa, Yutaka Inoue

**Affiliations:** 1 Faculty of Pharmacy and Pharmaceutical Sciences, Josai University, 1-1 Keyakidai, Sakado 3500295, Saitama, Japan; yy17116@josai.ac.jp (H.S.); sitakura@josai.ac.jp (S.I.); ht-todo@josai.ac.jp (H.T.); tanikawa@josai.ac.jp (T.T.); 2 Faculty of Science, Josai University, 1-1 Keyakidai, Sakado 3500295, Saitama, Japan; suzukoh@josai.ac.jp; 3 Pharmaceutical Research & Drug Development Laboratories, Department of Pharmacy, School of Health Care Professions, University of San Carlos, Cebu 6000, Philippines; fvarce@usc.edu.ph (F.A.J.); glsee@usc.edu.ph (G.L.S.); 4 Pharmaceutical Sciences Division, National Research Council of the Philippines, Taguig City 1631, Philippines

**Keywords:** ellagic acid, urea, complex formation, solubility, antioxidant capacity

## Abstract

Ellagic acid (EA), a natural polyphenol found in berries, has high antioxidant capacity. This study aimed to improve EA solubility by complex formation with urea (UR) using solvent evaporation method and evaluate its solubility, antioxidant capacity, and physical properties. The solubility test (25 °C, 72 h) showed that the solubility of EVP (EA/UR = 1/1) was approximately two-fold higher than that of EA (7.13 µg/mL versus 3.99 µg/mL). Moreover, the IC_50_ values of EA and EVP (EA/UR = 1/1) (1.50 µg/mL and 1.30 µg/mL, respectively) showed higher antioxidant capacity of EVP than that of EA. DSC analysis revealed that the UR peak at 134 °C disappeared, and a new endothermic peak was observed at approximately 250 °C for EVP (EA/UR = 1/1). PXRD measurements showed that the characteristic peaks of EA at 2θ = 12.0° and 28.0° and of UR at 2θ = 22.0°, 24.3°, and 29.1° disappeared and that new peaks were identified at 2θ = 10.6°, 18.7°, and 26.8° for EVP (EA/UR = 1/1). According to 2D NOESY NMR spectroscopy, cross-peaks were observed between the -NH and -OH groups, suggesting intermolecular interactions between EA and UR. Therefore, complexation was confirmed in EA/UR = 1/1 prepared by solvent evaporation, suggesting that it contributed to the improvement in solubility and antioxidant capacity of EA.

## 1. Introduction

Ellagic acid (EA) is a naturally occurring polyphenol widely distributed in berries, including strawberry, pomegranate, and nuts [1]. The absorption of EA, having four phenolic hydroxyl groups, occurs by hydrolysis in the intestine after oral ingestion of ellagitannins, a class of hydrolyzable tannins [2]. EA is a free radical scavenger and has high antioxidant activity; its whitening efficacy is generally attributable to inhibiting the proliferation of melanin-forming cells and melanin synthesis by tyrosinase [3,4,5]. Moreover, it has been reported to have anti-inflammatory, anticarcinogenic, and antimutagenic properties [6,7,8]. Nevertheless, EA is poorly soluble in water (less than 1 µg/mL) as well as in organic solvents, resulting in insufficient drug absorption [9]. This characteristic is an obstacle to the development of products containing EA; hence, improvement is required.

Urea (UR) has moisturizing properties; hydrates the stratum corneum; has keratin cleavage properties; and is commonly used as a moisturizer, skin permeability enhancer, and solubilizer in pharmaceuticals and cosmetics [10,11]. UR is known to have a hydrotropic effect [12]. This hydrotropic effect has been used for the solubilization of pharmaceuticals, for example, it has been reported to improve the solubility of diclofenac, a nonsteroidal anti-inflammatory drug [13]. UR generally exists as a polymorph with tetragonal and hexagonal crystal structures in the solid state. UR, in a hexagonal crystal structure, has been reported to improve solubility by enclosing the guest molecules within its pores [14]. It has also been shown that UR can form co-crystals with drugs such as benzamide and complex with palmitic acid [15,16]. Thus, UR is an interesting additive in the manufacture of pharmaceutical materials. Co-crystallization is believed to improve the solubility and bioavailability of the active ingredient without modifying its chemical structure because the properties of the second component (additive) incorporated into the crystal structure are reflected in the solid state [17]. In particular, the co-crystals of resveratrol and nicotinamide have been reported to improve solubility, and the co-crystals of theophylline and oxalic acid have been reported to improve stability [18,19].

UR can contribute to an improvement in solubility and stability, thereby adding new properties to pharmaceutical products. In this study, we aimed to improve the antioxidant capacity and solubility of EA though the preparation of a complex with UR by evaporation.

## 2. Materials and Methods

### 2.1. Materials

**Figure 1:**
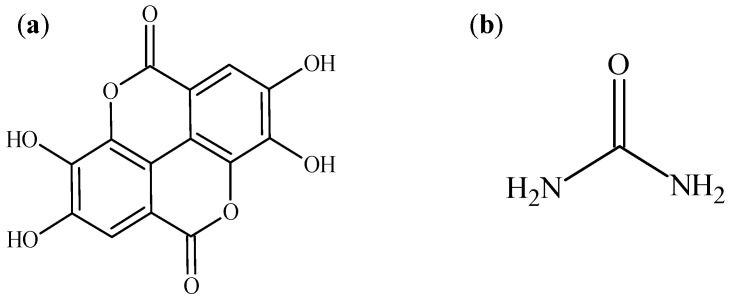
(**a**) Ellagic acid (EA); (**b**) Urea (UR).

EA was purchased from Cosmo Bio Co., Ltd. (lot number 284224, LKT Laboratories Inc., St Paul, MN, USA) (Figure 1a). UR (lot number 213-00173) and other reagents of commercial grade were supplied by FUJIFILM Wako Pure Chemical Corporation (Tokyo, Japan) (Figure 1b). NMR solvent was supplied by FUJIFILM Wako Pure Chemical Corporation (Cambridge Isotope Laboratories, Inc., Tewksbury, MA, USA).

### 2.2. Methods

#### 2.2.1. Preparation of Physical Mixture and Solvent Evaporated Solid Dispersion

The physical mixture (PM) was prepared by mixing EA (100 mg) and UR (19.78 mg) in 1:1 molar ratio using a vortex mixer for 1 min. The solvent evaporated solid dispersion (EVP; EA/UR = 1/1) was prepared by dissolving PM in 100 mL of ethanol, followed by evaporation (Rotavapor R-215; Buchi Labortechnik AG, Flawil, Switzerland) at 40 °C and 58 mbar.

#### 2.2.2. Solubility Test

In this study, a solubility test was performed to determine the solubility of EA/UR in distilled water. Each of EA, PM (EA/UR = 1/1), and EVP (EA/UR = 1/1) samples (equivalent to 10 mg of EA) were added individually to 10 mL distilled water and shaken in a thermostatic incubator (Bio Shaker BR-42FL; TAITEC, Saitama, Japan) at 25 °C and 100 rpm for 1, 3, 6, 24, and 72 h. The test solution was filtered through a 0.2 µm membrane filter (Advantec^®^, Toyo Roshi Kaisha, Ltd., Tokyo, Japan), diluted to 5–10 mL with ethanol, and quantified for its drug content using high-performance liquid chromatography (HPLC; Waters, Alliance System, e2695). The column used was Cosmosil 5C18AR-II ODS-3 (4.6 × 150 mm, 5 µm diameter, lot no K51712) at a temperature of 40 °C; the sample injection volume was 50 µL. The mobile phase was prepared as a mixture of 0.1% phosphoric acid/acetonitrile (4/1), EA retention time was set to 7 min, and the measurement wavelength was 254 nm (Waters, Alliance System, 2489).

#### 2.2.3. Dissolution Test

Dissolution test was performed using NTR-593 dissolution apparatus (Toyama Sangyo Co., Ltd., Tokyo, Japan) according to the JP XVII revised dissolution test using the paddle method. The weighed amount of sample (equivalent to 1.8 mg of EA) was placed in 900 mL distilled water at 37 ± 0.5 °C and stirred at 100 rpm. Aliquots (5 mL) of dissolved samples were collected at 5, 10, 15, 30, and 60 min and filtered through a 0.2 µm membrane filter (Advantec^®^, Toyo Roshi Kaisha, Ltd., Tokyo, Japan). After sample collection, the same amount of solution was added at the same temperature to maintain a constant volume of the dissolution medium. Quantification was performed by HPLC, as mentioned in the Section 2.2.2.

#### 2.2.4. DPPH Radical Scavenging Test

The DPPH radical scavenging test was performed to evaluate the antioxidant capacity of EA, PM (EA/UR = 1/1), and EVP (EA/UR = 1/1). Ascorbic acid (ASC) was used as a reference compound. One hundred microliters of 2,2-diphenyl-1-picrylhydrazyl (DPPH; 50 µM in ethanol) solution and samples at different concentrations were added to microplates at a volume ratio of 1/1. Ethanol (100 µL) was used as the control. The microplates were incubated for 5 min at 37 °C in the dark, and the absorbance was measured at a wavelength of 517 nm using the SpectraMax microplate reader (Molecular Devices Co., San Jose, CA, USA). Radical scavenging activity was calculated using Equation (1) [20].

Radical scavenging rate = [1 − (*A*_s_ − *Bl*)/(*A*_0_ − *Bl*)] × 100 (1)

*A*_s_—absorbance of sample; *A*_0_—absorbance without tested sample; and *Bl*—blank.

#### 2.2.5. Differential Scanning Calorimetry (DSC) Measurement

DSC measurements were performed using Thermo Plus EVO high-sensitivity differential scanning calorimeter (Rigaku, Tokyo, Japan). Approximately 2 mg of each sample was placed in a sealed aluminum pan, and the temperature was increased at a rate of 10 °C/min under nitrogen gas flow at 60 mL/min.

#### 2.2.6. Powder X-ray Diffraction (PXRD) Measurement

PXRD measurements were performed using the MiniFlex II PXRD system (Rigaku, Tokyo, Japan). The diffraction intensity was measured using a NaI scintillation counter. PXRD was performed using Cu Kα radiation (30 kV, 15 mA), with a scan rate of 4°/min and a scan range of 5–40° (2θ). The powder samples were placed on top flat surface of glass plates.

#### 2.2.7. Solid-State Fluorescence Measurement

Solid-state fluorescence spectra were obtained using FP-770F fluorescence spectrophotometer (JASCO Ltd., Tokyo, Japan). The powdered sample was placed in a front-reflecting cell (FP-1060) at an excitation wavelength of 388 nm, an excitation and fluorescence bandwidth of 5 nm, and a measurement range of 350–650 nm.

#### 2.2.8. Fourier Transform Infrared (FT-IR) Absorption Spectrum

The FT-IR spectra were obtained by the potassium bromide (KBr) pellet method using FT/IR-460 Plus (JASCO) with an integration frequency of 32 times, a resolution of 4 cm^−1^, and a wavenumber range of 4000–650 cm^−1^. The tablets were prepared by mixing the sample with KBr at a weight ratio of 1/10 and then pressing manually. Background correction was performed using KBr alone.

#### 2.2.9. Near-Infrared (NIR) Measurement

NIR spectra were obtained using Fourier transform near-infrared spectrometer (Buchi NIRFlex N-500; Nihon Buchi, Tokyo, Japan); samples were filled in sample cups and measured at a wavelength of 1000–4000 cm^−1^, time of 8 s, temperature of 25 °C, and optical path of 1 nm.

#### 2.2.10. Scanning Electron Microscopy (SEM)

SEM measurements were performed using S3000N SEM (Hitachi High-Technologies, Tokyo, Japan) at an accelerating voltage of 15 kV. The vacuum gold-steaming time of the samples was 70 s.

#### 2.2.11. Nuclear Overhauser Effect Spectroscopy (NOESY) Measurement

Nuclear magnetic resonance (NMR) spectroscopy analysis was performed using Varian 700 MHz NMR spectrometer (Varian NMR System 700NB; Agilent) with an HCN probe operating at 699.7 MHz and DMSO-d_6_ as solvent. Other conditions were as follows: mixing time, 1500 ms; waiting time 1 s; integration frequency, 256; and temperature, 25 °C.

#### 2.2.12. Statistical Analysis

The data are expressed as the mean ± standard deviation (S.D.). Tukey’s multiple comparison method was used in one-way analysis of variance (ANOVA) to compare differences between experimental groups; statistical significance was set at *p* < 0.01.

## 3. Results and Discussion

### 3.1. Solubility Test

We determined the solubility of EA, PM (EA/UR = 1/1), and EVP (EA/UR = 1/1). The solubility of EA was approximately 1.36 µg/mL at 3 h, 3.77 µg/mL at 24 h, and 3.99 µg/mL at 72 h (Table 1). The corresponding solubility of PM (EA/UR = 1/1) was approximately 2.06 µg/mL, 5.18 µg/mL, and 5.04 µg/mL. The solubility of PM was slightly higher than that of EA, which could be attributed to the hydrotropic property of UR [11]. On the other hand, the solubility of EVP (EA/UR = 1/1) was approximately 5.40 µg/mL at 3 h, 7.45 µg/mL at 24 h, and 7.13 µg/mL at 72 h, which was approximately 4-fold (at 3 h) and 2-fold (at 24 h and 72 h) higher than that of EA. This was attributable to the hydrotropic effect of UR and interaction between EA and UR.

### 3.2. Dissolution Test

**Figure 2:**
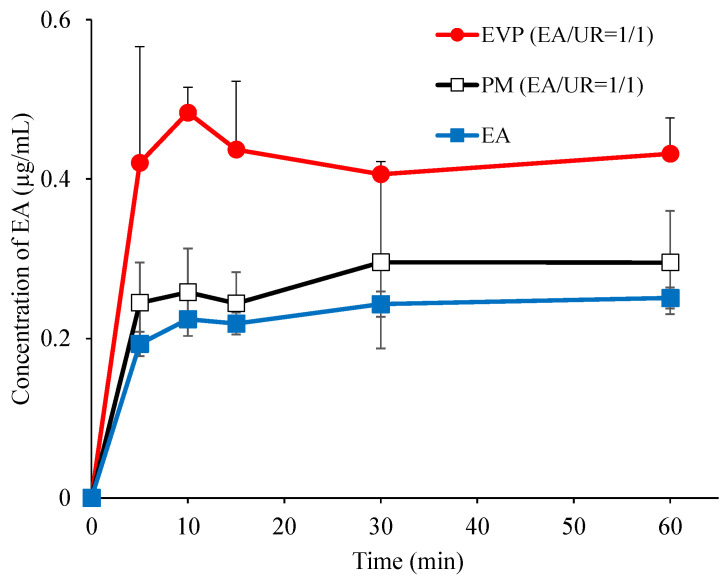
Dissolution profiles of EA/UR systems. Results are expressed as the mean ± S.D. (n = 3).

Dissolution studies were conducted to assess the dissolution behavior of EA, PM (EA/UR = 1/1), and EVP (EA/UR = 1/1) (Figure 2). The concentration of EA was 0.19 µg/mL and 0.24 µg/mL, respectively, in EA and PM (EA/UR = 1/1) in 5 min-aliquots, whereas it was 0.42 µg/mL in EVP (EA/UR = 1/1) (2-fold that of EA and PM). Therefore, results suggested that solid-state interactions contributed to the improved dissolution profile of EVP (EA/UR = 1/1).

### 3.3. DPPH Radical Scavenging Test

**Figure 3:**
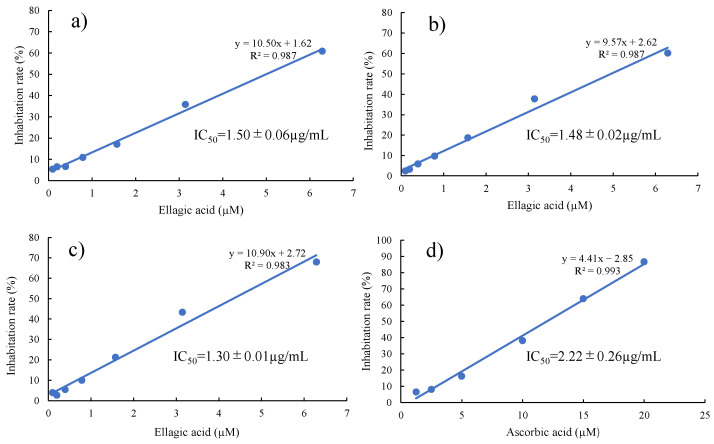
DPPH radical scavenging activity of EA/UR systems. Results are expressed as the mean ± S.D. (n = 3). (**a**) EA; (**b**) PM (EA/UR = 1/1); (**c**) EVP (EA/UR = 1/1); and (**d**) ASC.

DPPH radical scavenging assay of EVP (EA/UR = 1/1) was performed to investigate whether its improved solubility and dissolution contributed to the antioxidant effect (Figure 3). ASC was used as a reference compound for comparison. The IC50 of EA (approximately 1.50 µg/mL) was significantly lower than that of ASC (approximately 2.22 µg/mL). The IC50 of PM (EA/UR = 1/1) was 1.48 µg/mL, which was similar to that of EA, whereas the IC50 of EVP (EA/UR = 1/1) was 1.30 µg/mL, which was lower than that of EA. Inoue et al. reported that daidzein, an additive and antioxidant, exhibits antioxidant effect owing to the intermolecular interactions caused by complex formation with cyclodextrin [21]. Hence, it was assumed that the increased radical scavenging activity of EVP (EA/UR = 1/1) was attributable to the increase in electron density in the complex of EA and UR, releasing more protons as radicals. Thus, EA/UR complex formation may improve the solubility and antioxidant capacity of EA.

### 3.4. DSC Measurement

**Figure 4:**
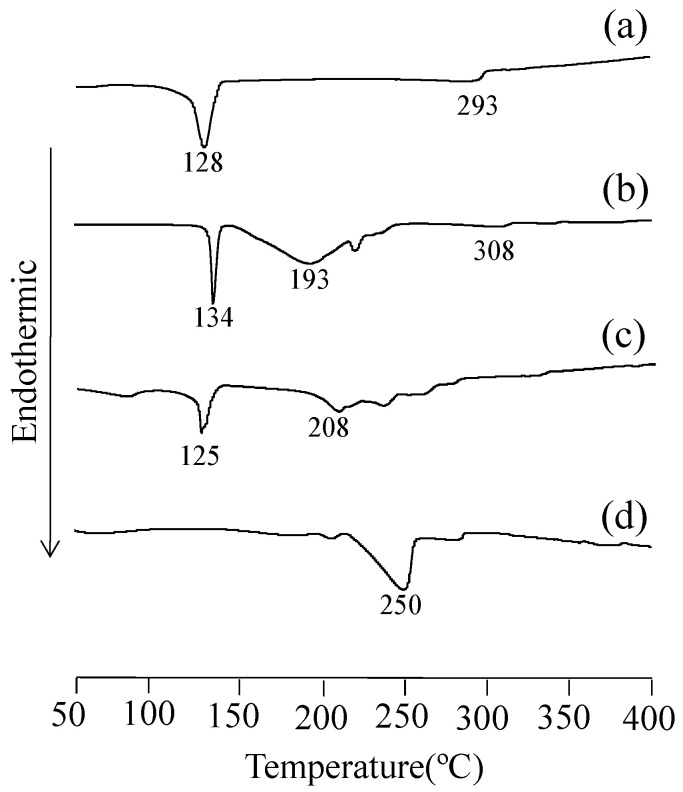
DSC curves of EA/UR systems. (**a**) EA; (**b**) UR; (**c**) PM (EA/UR = 1/1); and (**d**) EVP (EA/UR = 1/1).

DSC was performed to investigate the thermal behavior of the EA/UR complex in the solid state that showed improved solubility and antioxidant capacity (Figure 4). For EA, an endothermic peak, attributed to the incorporation of water of crystallization, was observed at approximately 128 °C. In addition, a melting peak was observed at approximately 293 °C. For UR, an endothermic peak (melting) was observed at approximately 134 °C. For PM (EA/UR = 1/1), and a broad peak due to the melting of UR and water of crystallization was observed at approximately 125 °C. In contrast, for EVP (EA/UR = 1/1), the endothermic peaks due to the melting of EA and UR disappeared, and a new peak at approximately 250 °C was observed. These findings suggest that EVP (EA/UR = 1/1) prepared by evaporation of EA and UR resulted in a thermally stable complex formation.

### 3.5. PXRD Measurement

**Figure 5:**
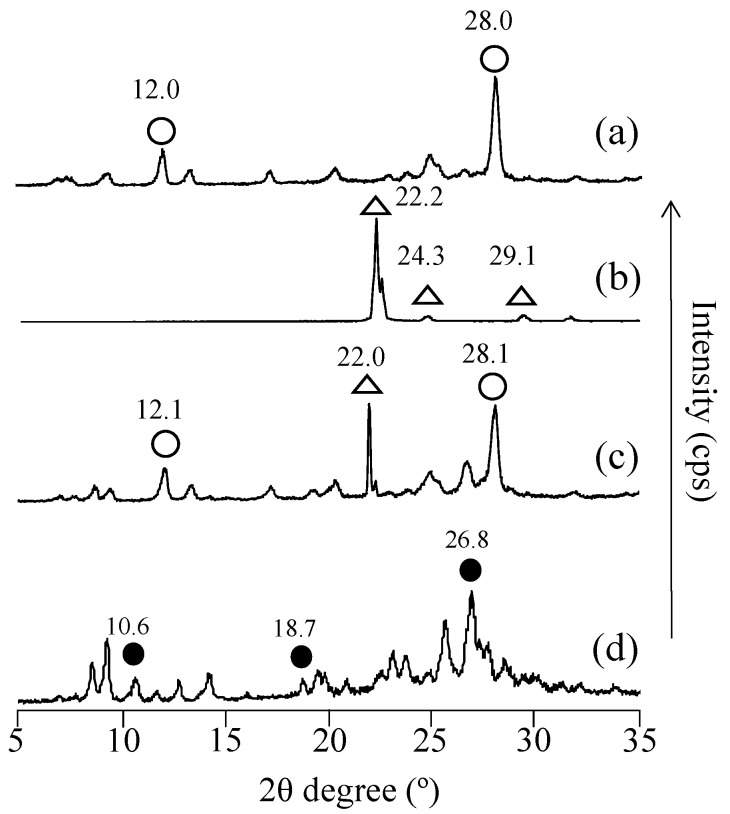
PXRD patterns of EA/UR systems. (**a**) EA; (**b**) UR; (**c**) PM (EA/UR = 1/1); and (**d**) EVP (EA/UR = 1/1). ○ EA, △ UR, and ● new peak.

As complex formation was confirmed by DSC, PXRD analysis was performed to observe changes in the crystal structure (Figure 5). In EA, characteristic peaks (2θ) were observed at 12.0° and 28.0°, whereas in UR, characteristic diffraction peaks (2θ) were observed at 22.2°, 24.3°, and 29.1°. In PM (EA/UR = 1/1), characteristic EA-derived diffraction peaks (2θ) were observed at 12.1° and 28.2°, and the UR-derived diffraction peak was observed at 22.0°. In contrast, in EVP (EA/UR = 1/1), characteristic diffraction peaks of EA and UR disappeared, and new peaks were observed at 2θ = 10.6°, 18.7°, and 26.8°. This suggests the possibility of a complex formation between EA and UR in EVP (EA/UR = 1/1). However, in EVP (EA/UR = 2/1 and 1/2), EA-derived peaks remained at 2θ = 12.0° and 28.0°, and UR-derived peaks remained at 2θ = 22.2°, 24.6°, and 29.2°, suggesting the possibility of complex formation in only EVP (EA/UR = 1/1) (Figure S1). The DSC and PXRD results suggest that the complex of EA and UR was formed at a molar ratio of 1/1.

### 3.6. Solid Fluorescence Measurement

**Figure 6:**
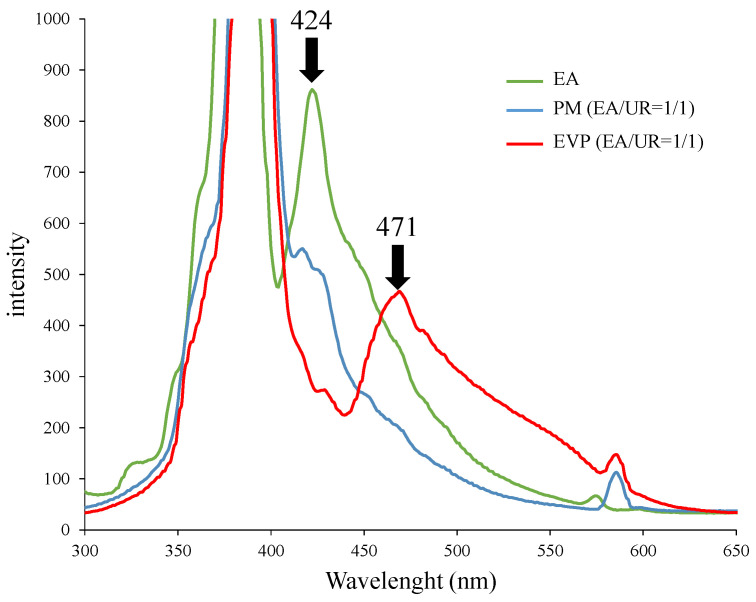
Fluorescence spectra of EA/UR systems.

Solid-state fluorescence spectroscopy was conducted to determine changes in the molecular state due to complex formation between EA and UR (Figure 6). A peak at 424 nm was observed for EA, and a shoulder peak at 420 nm was observed for PM (EA/UR/ = 1/1). In contrast, in EVP (EA/UR = 1/1), the EA peak at 424 nm was reduced, and a new peak at approximately 471 nm was observed. In solid-state fluorescence measurements, changes in the molecular state were observed in conjunction with changes in the fluorescence spectrum [22]. This indicated that molecular state of the aromatic ring of EA was distorted by its interaction with UR, which might have affected the π–π bond.

### 3.7. FT-IR Absorption Spectrum

**Figure 7:**
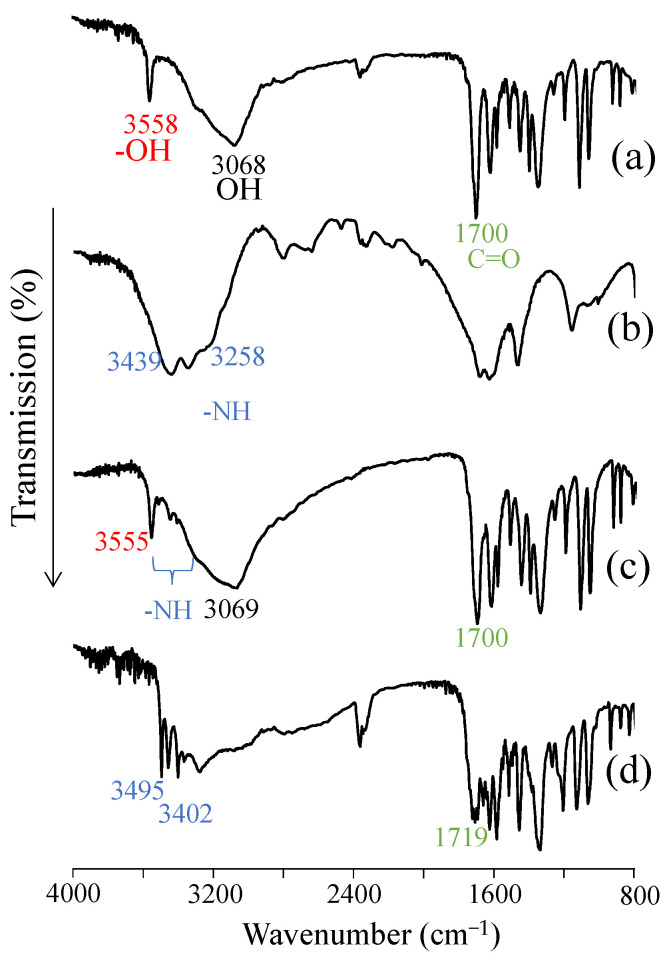
FT-IR absorption spectra of EA/UR systems. (**a**) EA; (**b**) UR; (**c**) PM (EA/UR = 1/1); and (**d**) EVP (EA/UR = 1/1).

Solid-state fluorescence measurements suggested that the solid molecular state of EA was affected during complex formation with UR. Therefore, FT-IR was used to confirm the intermolecular state in the complex of EA and UR (Figure 7). The peak related to the phenolic hydroxyl group in EA was observed at approximately 3558 cm^−1^. In UR, peaks were observed at 3439 cm^−1^ and 3258 cm^−1^ and were attributed to the -NH group. In PM (EA/UR = 1/1), peaks at approximately 3556 cm^−1^ (derived from the hydroxyl group of EA) and 1700 cm^−1^ (derived from the C=O group of EA) were observed [23]. Meanwhile, in EVP (EA/UR = 1/1), the hydroxyl group peak of EA disappeared, and the -NH group peak of UR shifted to 3495 cm^−1^ and 3402 cm^−1^. Furthermore, the C=O group peak of EA at 1700 cm^−1^ shifted to 1719 cm^−1^, suggesting that the intermolecular interaction of the -OH group observed in EA alone was dissociated, and hydrogen bonding between the -OH and C=O groups of EA and the -NH group of UR was inferred. However, it was difficult to distinguish the -NH group from the -OH group; thus, NIR measurements were performed.

### 3.8. NIR Measurement

**Figure 8:**
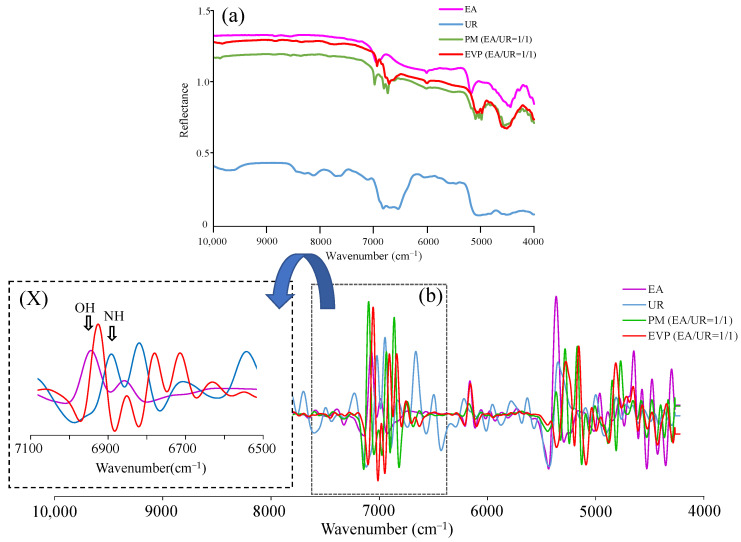
(**a**) NIR absorption spectra of EA/UR systems observed at 4000–10,000 cm^−1^; (**b**) second derivative NIR absorption spectra of EA/UR systems observed at 4000–10,000 cm^−1^; and (**X**) second derivative NIR absorption spectra of EA/UR systems observed at 6500–7100 cm^−1^.

Although intermolecular interaction between the -OH group of EA and the -NH group of UR was speculated from FT-IR results, NIR measurement was performed because it was difficult to distinguish the -NH group from the -OH group (Figure 8). The peak of the -OH group at 6960 cm^−1^ in EA shifted to 6932 cm^−1^ in EVP (EA/UR = 1/1). The peaks of the -NH group in UR observed at 6896 cm^−1^, 6820 cm^−1^, and 6700 cm^−1^ shifted to 6780 cm^−1^, 6704 cm^−1^, and 6620 cm^−1^ in EVP (EA/UR = 1/1). These results suggested intermolecular interactions by hydrogen bonding between the -OH group of EA and the -NH group of UR. The FT-IR results also suggested intermolecular interactions between the -NH group of UR and the -OH group of EA and the aromatic ring; therefore, the results of NIR measurements correlate with the FT-IR results.

### 3.9. SEM Analysis

**Figure 9:**
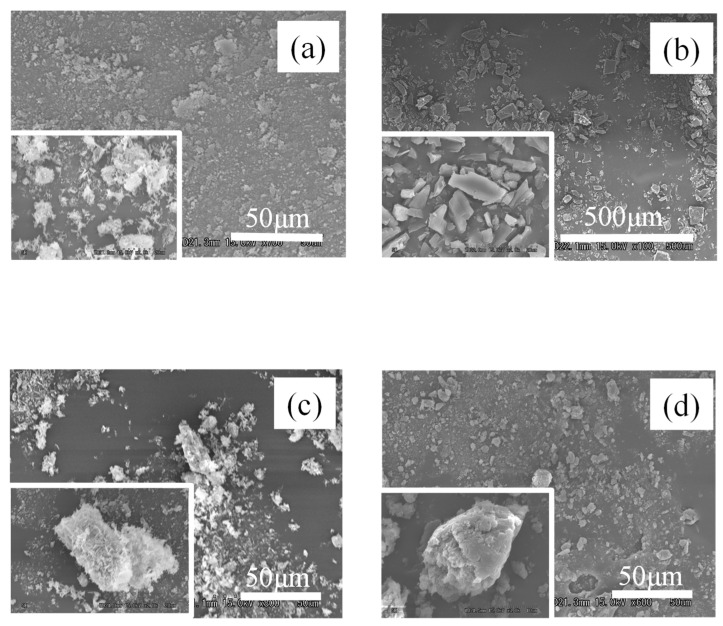
SEM analysis of EA/UR systems. (**a**) EA; (**b**) UR; (**c**) PM (EA/UR = 1/1); (**d**) EVP (EA/UR = 1/1).

SEM analysis was performed to observe morphological characteristics of the prepared sample (Figure 9). Powdery particles with a rough surface were observed in EA, and flat crystals were observed in UR. In reference to the SEM images of PM (EA/UR = 1/1), EA was attached to the crystal surface of UR. In contrast, the surface of EVP (EA/UR = 1/1) was smoother than that of PM (EA/UR = 1/1), and ellipsoidal crystals were observed. It was inferred that the complex, which was formed by intermolecular interactions between EA and UR during evaporation, was identified in the solid state, and large specific surface area of EVP (EA/UR = 1/1) might have contributed to the improved solubility.

### 3.10. NOESY NMR Measurement

**Figure 10:**
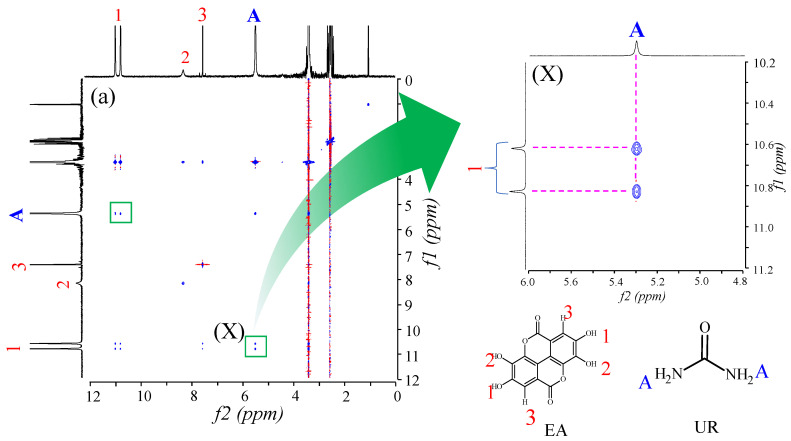
NOESY NMR spectra of EA/UR systems in DMSO-d6. (**a**) f1, 0–12 ppm; f2, 0–12 ppm (**X**); f1, 10.2–11.2 ppm; and f2, 4.8–6.0 ppm.

**Scheme 1:**
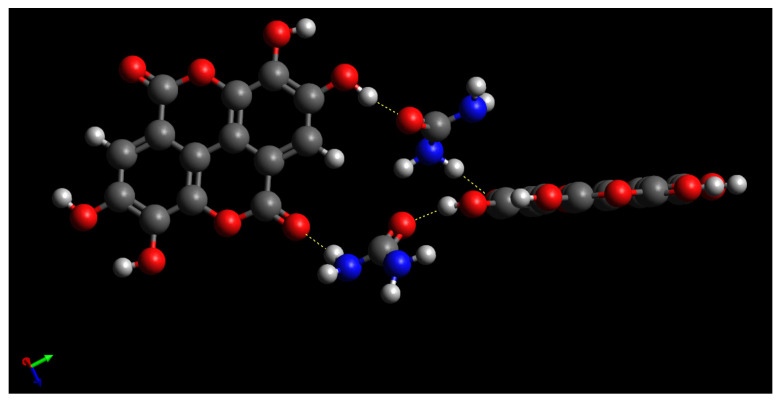
Proposed structure of the EA/UR complex.

2D-NOESY NMR measurements were performed to observe intermolecular interactions of EVP (EA/UR = 1/1) in solution (Figure 10). A cross-peak was observed between 7.45 ppm derived from the -NH group of EA and 10.62 and 10.83 ppm derived from the -OH group of EA, suggesting intermolecular interactions between the -OH group of EA and the -NH group of UR. Interestingly, the complex formation between EA and UR was also observed in the solution, indicating its contribution to the improved solubility of EVP (EA/UR = 1/1). Fucheng et al. reported EA/UR complex formation at a molar ratio of 1/2 [24]. However, in this study, EA/UR complex was formed at a molar ratio of 1/1 by solvent removal; further, the PXRD pattern of EA/UR (molar ratio = 1/2) reported by Fucheng et al. differed from that of EA/UR (molar ratio = 1/1) in this study. Therefore, we fabricated an EA/UR = 1/1 complex by solvent evaporation, the results of which suggest that the increased solubility and antioxidant capacity are attributable to the complexation of EA and UR in solution, which increases the electron density of the aromatic ring of EA and facilitates proton dissociation. The predicted interactions of the EA/UR complex are shown in Scheme 1.

## 4. Conclusions

In the present study, the EA/UR complex was formed at a molar ratio of 1/1 by solvent evaporation. EVP (EA/UR = 1/1) showed the improved solubility and antioxidant capacity of EA. The IC_50_ values of EA and EVP (EA/UR = 1/1) (1.50 µg/mL and 1.30 µg/mL, respectively) showed the higher antioxidant capacity of EVP than that of EA. DSC analysis revealed that the UR peak at 134 °C disappeared, and a new endothermic peak was observed at approximately 250 °C for EVP (EA/UR = 1/1). The improved antioxidant capacity of the complex of EA and UR at a molar ratio of 1/1 prepared using the solvent evaporation method has potential for skin application. 

## Figures and Tables

**Table 1:** Solubility profiles of EA/UR systems in distilled water at 25 °C.

Time	EA	PM	EVP
3	1.36 ± 0.26	2.06 ± 0.69	5.40 ± 2.27
24	3.77 ± 0.36	5.18 ± 0.30	7.45 ± 1.65
72	3.99 ± 0.59	5.04 ± 1.33	7.13 ± 0.49

Results are presented as the mean ± S.D. (n = 3).

## Data Availability

Not applicable.

## Supplementary Materials

The following supporting information can be downloaded at: https://www.mdpi.com/1996-1944/15/8/2836/s1, Figure S1: PXRD patterns of EA/UR systems. (a) EA; (b) UR; (c) PM (EA/UR = 1/2); (d) PM (EA/UR = 2/1); (e) EVP (EA/UR = 1/2); (f) EVP (EA/UR = 2/1).

## Abbreviations

EA	Ellagic acid
UR	Urea
PM	Physical mixture
EVP	Evaporated
ASC	Ascorbic acid
DPPH	2,2-Diphenyl-1-picrylhydrazyl
PXRD	Powder X-ray diffraction
DSC	Differential scanning calorimetry
FT-IR	Fourier transform infrared spectroscopy
NIR	Near-infrared
SEM	Scanning electron microscopy
NOESY	Nuclear Overhauser effect spectroscopy

## References

[1] Ceci C., Lacal P.M., Tentori L., Gabriella De Martino M., Miano R., Graziani G. (2018). Experimental evidence of the antitumor, antimetastatic and antiangiogenic activity of ellagic acid. Nutrients.

[2] Theocharis G., Andlauer W. (2013). Innovative microwave-assisted hydrolysis of ellagitannins and quantification as ellagic acid equivalents. Food Chem..

[3] Seeram N.P., Lee R., Heber D. (2004). Bioavailability of ellagic acid in human plasma after consumption of ellagitannins from pomegranate (*Punica granatum* L.) juice. Clin. Chim. Acta.

[4] Yoshimura M., Watanabe Y., Kasai K., Yamakoshi J., Koga T. (2005). Inhibitory effect of an ellagic acid-rich pomegranate extract on tyrosinase activity and ultraviolet-induced pigmentation. Biosci. Biotechnol. Biochem..

[5] Mertens-Talcott S.U., Talcott S.T., Percival S.S. (2003). Low concentrations of quercetin and ellagic acid synergistically influence proliferation, cytotoxicity and apoptosis in MOLT-4 human leukemia cells. J. Nutr..

[6] Marín M., Giner R.M., Ríos J.-L., Recio M.C. (2013). Intestinal anti-inflammatory activity of ellagic acid in the acute and chronic dextrane sulfate sodium models of mice colitis. J. Ethnopharmacol..

[7] Zheng W., Wang S.Y. (2001). Antioxidant activity and phenolic compounds in selected herbs. J. Agric. Food Chem..

[8] Castonguay A., Boukharta M., Teel R. (1998). Biodistribution of, antimutagenic efficacies in Salmonella typhimurium OF, and inhibition of P450 activities by ellagic acid and one analogue. Chem. Res. Toxicol..

[9] Nyamba I., Lechanteur A., Semdé R., Evrard B. (2021). Physical formulation approaches for improving aqueous solubility and bioavailability of ellagic acid: A review. Eur. J. Pharm. Biopharm..

[10] Mueller J., Oliveira J.S.L., Barker R., Trapp M., Schroeter A., Brezesinski G., Neubert R.H.H. (2016). The effect of urea and taurine as hydrophilic penetration enhancers on stratum corneum lipid models. Biochim. Biophys. Acta.

[11] Trommer H., Neubert R.H.H. (2006). Overcoming the stratum corneum: The modulation of skin penetration. Skin Pharmacol. Physiol..

[12] Herbig M.E., Evers D.-H. (2013). Correlation of hydrotropic solubilization by urea with logD of drug molecules and utilization of this effect for topical formulations. Eur. J. Pharm. Biopharm..

[13] Takahashi K., Suzuki T., Sakano H., Mizuno N. (1955). Effect of vehicles on diclofenac permeation across excised rat skin. Biol. Pharm. Bull..

[14] Inoue Y., Niiyama D., Murata I., Kanamoto I. (2017). Usefulness of urea as a means of improving the solubility of poorly water-soluble ascorbyl palmitate. Int. J. Med. Chem..

[15] Beig A., Lindley D., Miller J.M., Agbaria R., Dahan A. (2016). Hydrotropic solubilization of lipophilic drugs for oral delivery: The effects of urea and nicotinamide on carbamazepine solubility-permeability interplay. Front. Pharmacol..

[16] Cysewski P., Przybylek M., Ziolkowska D., Mroczynska K. (2016). Exploring the cocrystallization potential of urea and benzamide. J. Mol. Model..

[17] Fukami T. (2017). Current status and promising future of pharmaceutical cocrystals in development of oral dosage forms. Nippon Yakurigaku Zasshi.

[18] Yanez M., Jhanji M., Murphy K., Gower R.M., Sajish M., Jabbarzadeh E. (2019). Nicotinamide augments the anti-inflammatory properties of resveratrol through PAPR1 activation. Sci. Rep..

[19] Trask A.V., Motherwell W.D.S., Jones W. (2006). Physical stability enhancement of theophylline via cocrystallization. Int. J. Pharm..

[20] Chao J., Wang H., Zhao W., Zhang M., Zhang L. (2012). Investigation of the inclusion behavior of chlorogenic acid with hydroxypropyl-β-cyclodextrin. Int. J. Biol. Macromol..

[21] Inoue Y., Yoshida M., Ezawa T., Tanikawa T., Arce F., See G.L., Tomita J., Suzuki M., Oguchi T. (2022). Inclusion complexes of daidzein with cyclodextrin-based metal-organic framework-1 enhance its solubility and antioxidant capacity. AAPS PharmSciTech..

[22] Brittain H.G., Elder B.J., Isbester P.K., Salerno A.H. (2005). Solid-state fluorescence studies of some polymorphs of diflunisal. Pharm. Res..

[23] Moribe K., Tsuchiya M., Tozuka Y., Yamaguchi K., Oguchi T., Yamamoto K. (2004). Grinding-induced equimolar complex formation between thiourea and ethenzamide. Chem. Pharm. Bull..

[24] Leng F., Robeyns K., Leyssens T. (2021). Urea as a cocrystal former–Study of 3 urea based pharmaceutical cocrystals. Pharmaceutics.

