# Supplementary figures and images for: Preparation, Characterization, Solubility, and Antioxidant Capacity of Ellagic Acid-Urea Complex
Source: Materials (Basel). 2022 Apr 12;15(8):2836. doi: 10.3390/ma15082836 (PMC9032788; doi:10.3390/ma15082836)

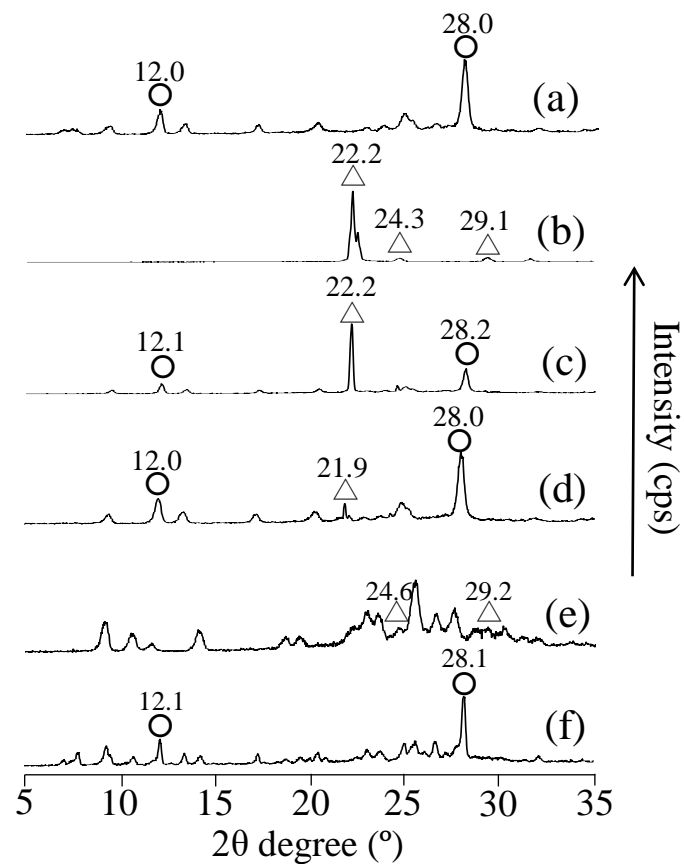

**Figure S1.** PXRD patterns of EA/UR systems.  
(a) EA; (b) UR; (c) PM (EA/UR=1/2); (d) PM (EA/UR=2/1); (e) EVP (EA/UR=1/2); (f) EVP (EA/UR=2/1).

Supplement: Supplementary file 1 [file materials-15-02836-s001.zip › materials-1646467-supplementary.pdf]
